# Anomalous origin of the left circumflex coronary artery from the pulmonary artery. A very rare congenital anomaly in an adult patient diagnosed by cardiovascular magnetic resonance

**DOI:** 10.1186/1532-429X-10-4

**Published:** 2008-01-21

**Authors:** Grigorios Korosoglou, Gerd Ringwald, Evangelos Giannitsis, Hugo A Katus

**Affiliations:** 1 Department of Cardiology, University of Heidelberg, Heidelberg, Germany

## Abstract

Here we report for the first time on the diagnostic potential of cardiovascular magnetic resonance (CMR) to delineate the proximal course of an anomalous left circumflex coronary artery (LCX) originating from the right pulmonary artery in an adult patient with no other form of congenital heart disease. The patient was referred to our institution due to exertional chest discomfort. X-Ray coronary angiography showed a normal left anterior descending coronary artery (LAD) and right coronary artery (RCA), while the LCX was filled retrograde by collateral flow through the LAD and the RCA. The origin of the LCX was postulated to be the pulmonary artery, but the exact origin of the anomalous artery could not be depicted on conventional angiograms. CMR provided the unambiguous depiction of the origin of the anomalous LCX from the right pulmonary artery and the delineation of its proximal course in this case of a very rare coronary anomaly in adults.

## 1. Introduction

This report describes the anomalous origin of the left circumflex coronary artery (LCX) from the right pulmonary artery (PA) in a 54-year-old woman with no other form of congenital heart disease. Coronary anomalies are a group of congenital disorders with highly variable pathophysiological mechanisms and manifestations. The occurrence of coronary anomalies is 0.3–0.9% in patients without structural heart disease and significantly higher (3–36%) in patients with structural heart defects [1,2]. Due to the variable clinical presentation and prognosis of coronary anomalies depending on the proximal course of the anomalously arising coronary artery in relation to the great vessels, their early detection, and the exact delineation of their proximal course are crucial [3,4].

## 2. Case presentation

**Figure 1:**
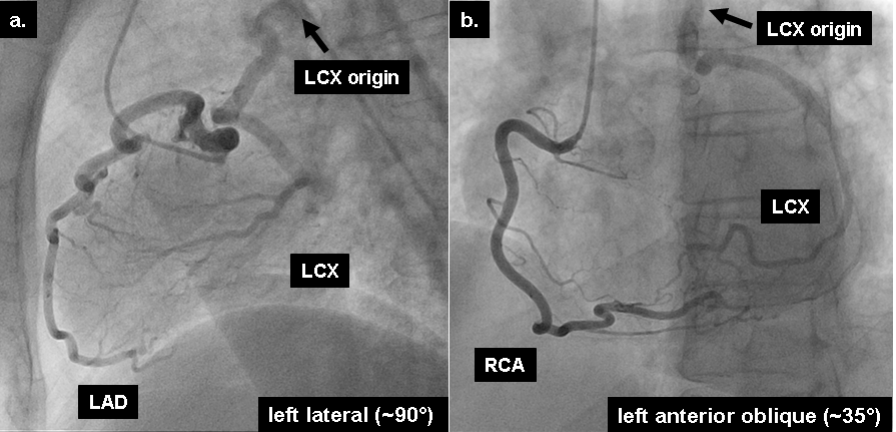
X-Ray coronary angiography shows the retrograde filling of an anomalous left circumflex artery (LCX) though collateral vessels provided by (a) the left anterior descending artery (LAD) and (b) the right coronary artery (RCA).

**Figure 2:**
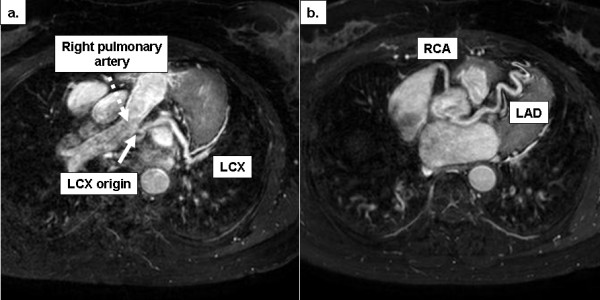
Multi-planar reformatted CMR images depict the exact origin of (a) the anomalous LCX from the right pulmonary artery and confirm the normal origin and course of (b) the LAD and RCA.

A 54-year-old woman was referred to our institution for routine coronary angiography due to exertional chest discomfort. An exercise ECG by her referring physician was clinically and electrically positive, demonstrating ST-segment depression in leads V5 and V6. Furthermore, nuclear scintigraphy had confirmed the presence of inducible ischemia in the lateral myocardial wall, by showing a reversible perfusion defect in this region during ergometric stress. Coronary angiography showed a normal left anterior descending coronary artery (LAD), while the LCX was filled retrogradely by collateral flow through the LAD (figure 1a). The right coronary artery (RCA) arose in typical position and also provided retrograde filling of the LCX (figure 1b). No evidence of atherosclerotic disease was found, either in the left system or in the RCA, and the origin of the LCX (black arrow) was postulated to be the pulmonary artery. To trace the exact anatomical origin of the anomalous LCX, cardiovascular magnetic resonance (CMR) was performed in a clinical 1.5T scanner (Achieva, Philips Medical Systems, Best, The Netherlands). CMR demonstrated normal function of the left ventricle (ejection fraction of 67%) and no signs of structural heart disease. Multi-planar reformatted images of T_1_-weighted, free-breathing, whole-heart acquisitions, allowed the unambiguous delineation of the proximal segments of all 3 coronary arteries. Thus, CMR provided the exact anatomical depiction of the origin of the anomalous LCX (solid arrow, figure 2a) from the right pulmonary artery (hatched arrow, figure 2a) and demonstrated the normal origin of the LAD and RCA (figure 2b). The patient was then referred for cardiac surgery.

## 3. Discussion

In summary, these findings highlight the role of CMR for detecting the exact origin and for delineating the proximal course of anomalous coronary arteries in a case of a very rare coronary anomaly in adults [5-7]. The anomalous origin of the LCX from the pulmonary artery is usually discovered in childhood, and is generally associated with other major congenital cardiac defects, such as patent ductus arteriosus, aortic coarctation, subaortic stenosis and pulmonary valve stenosis [8,9]. The presence of an anomalous LCX arising from the pulmonary artery in adults without congenital cardiac defects is very rare. The presentation in adults may be in the form of new-onset exertional angina, shortness of breath, abnormal ischemic changes on ECG or abnormal stress electrocardiography/nuclear scintigraphy [5-7]. Symptoms and prognosis are usually dependent upon the development of collateral vessels from the two other coronary arteries and the treatment is surgical with either ligation of the LCX at the origin alone, ligation with aorta-coronary bypass or reimplantation of the LCX to the aorta. Although conventional X-Ray angiography has traditionally been used to diagnose coronary anomalies, in some cases the exact orifice of anomalous coronary vessels cannot be selectively identified by this technique [6,10]. In our patient the origin of the LCX could not be determined on conventional angiograms and the exact diagnosis was established by the subsequent CMR scan. The three dimensional acquisitions of either CMR or computed tomography (CT) generally allow unambiguous interpretation of the locations of coronary origins. While CT generally offers better spatial resolution, the versatility of CMR can potentially provide additional information on the direction of flow in an anomalous vessel, and information on myocardial viability and perfusion, if required, all without ionizing radiation. However, in case of coronary anomalies coronary angiography should be performed in order to exclude additional atherosclerotic disease. Here we report for the first time on the diagnostic potential of CMR to delineate the proximal course of an anomalous LCX originating from the right pulmonary artery in an adult patient without structural heart disease.

## Competing interests

The author(s) declare that they have no competing interests.
